# Supplementary material for: Earthworms contribute significantly to global food production
Source: Nat Commun. 2023 Sep 26;14:5713. doi: 10.1038/s41467-023-41286-7 (PMC10522571; doi:10.1038/s41467-023-41286-7)
Supplement: Supplementary file 1 — Supplementary Information [file 41467_2023_41286_MOESM1_ESM.pdf]

Supplemental Information

Earthworms contribute significantly to global food production

Steven J. Fonte, Marian Hsieh and Nathaniel D. Mueller

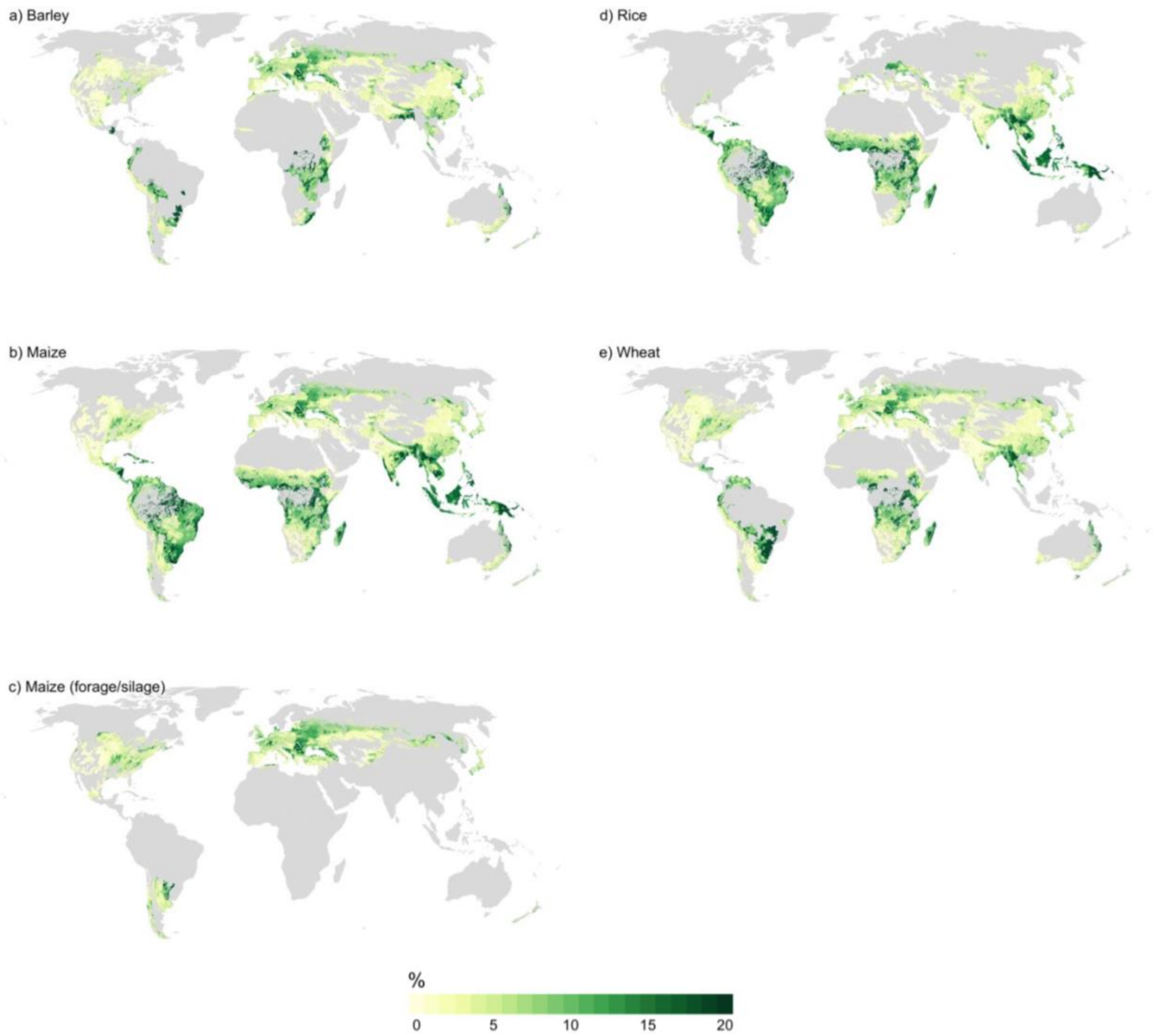

Figure S1: Relative contribution of earthworms to yield (% of total) for individual grain crops: a) barley, b) grain maize; c) silage maize, d) rice and e) wheat. Darker shades of green indicate stronger estimated earthworm impacts.

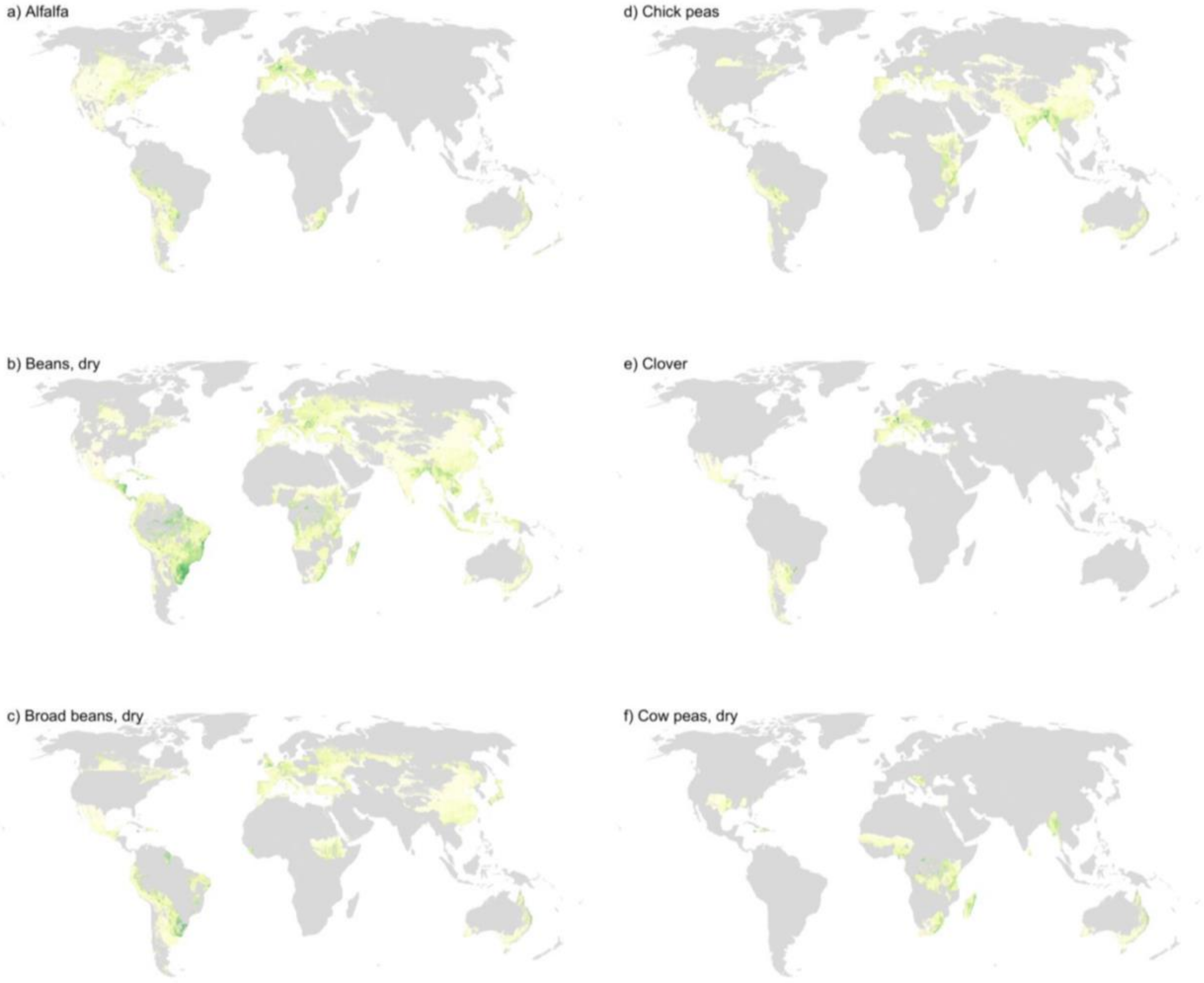
a) Alfalfa
d) Chick peas
b) Beans, dry
e) Clover
c) Broad beans, dry
f) Cow peas, dry

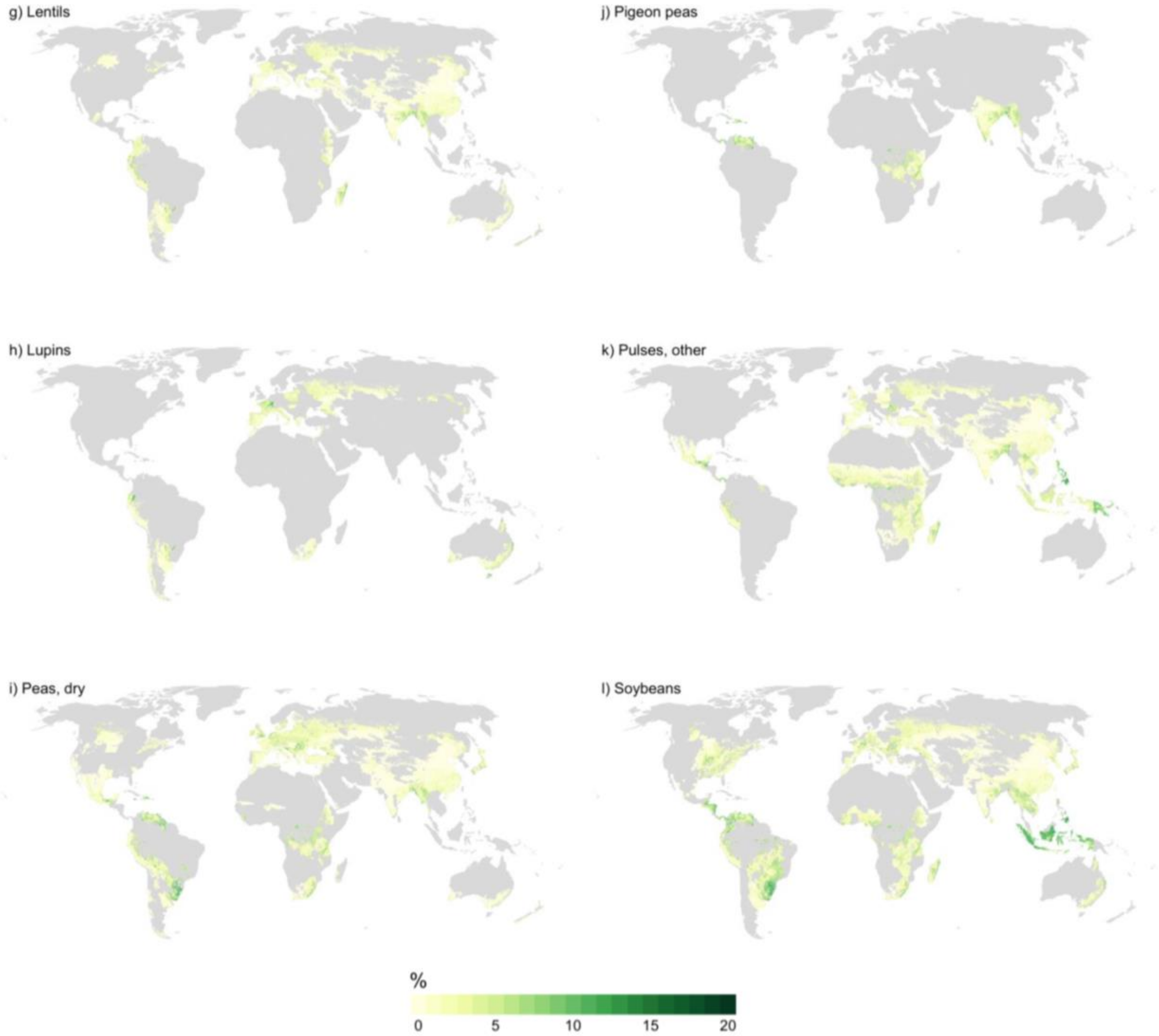

Figure S2: Relative contribution of earthworms to yield (% of total) of individual legume crops: a) alfalfa, b) dry beans, c) broad beans, d) chickpeas, e) clover, f) cowpea, g) lentils, h) lupins, i) peas, j) pigeon peas, k) other pulses, and l) soybean. Darker shades of green indicate stronger estimated earthworm impacts.

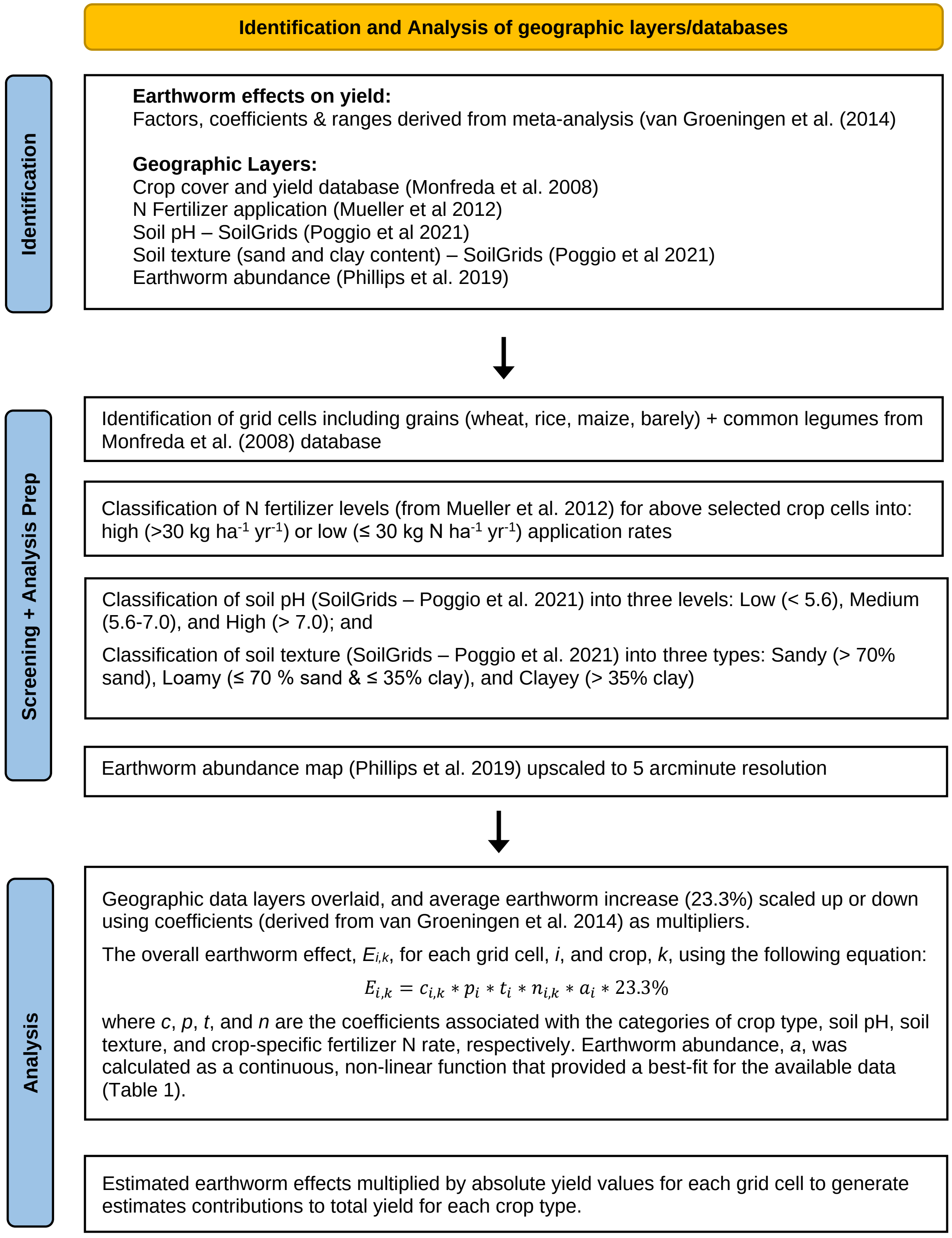

Figure S3: PRISMA flow diagram showing the workflow of data gathering, screening, and analysis applied in this study.

References:

1. van Groenigen, J. W. et al. Earthworms increase plant production: a meta-analysis. Sci. Rep. 4, 1-7 (2014).
2. Monfreda, C., Ramankutty, N. & Foley, J. A. Farming the planet: 2. Geographic distribution of crop areas, yields, physiological types, and net primary production in the year 2000. Glob. Biogeochem. Cycl. 22 (2008).
3. Mueller, N. D. et al. Closing yield gaps through nutrient and water management. Nature 490, 254-257 (2012).
4. Phillips, H. R. et al. Global distribution of earthworm diversity. Science 366, 480-485 (2019).
5. Poggio, L. et al. SoilGrids 2.0: producing soil information for the globe with quantified spatial uncertainty. SOIL 7, 217-240 (2021).
