# Supplementary material for: Earthworms contribute significantly to global food production
Source: Nat Commun. 2023 Sep 26;14:5713. doi: 10.1038/s41467-023-41286-7 (PMC10522571; doi:10.1038/s41467-023-41286-7)
Supplement: Supplementary file 2 — Reporting Summary [file 41467_2023_41286_MOESM2_ESM.pdf]

nature portfolio

Corresponding author(s): Steven Fonte

Last updated by author(s): 31 March 2023

Reporting Summary

Nature Portfolio wishes to improve the reproducibility of the work that we publish. This form provides structure for consistency and transparency in reporting. For further information on Nature Portfolio policies, see our Editorial Policies and the Editorial Policy Checklist.

Statistics

For all statistical analyses, confirm that the following items are present in the figure legend, table legend, main text, or Methods section.

n/a	Confirmed	
☐	☒	The exact sample size (*n*) for each experimental group/condition, given as a discrete number and unit of measurement
☒	☐	A statement on whether measurements were taken from distinct samples or whether the same sample was measured repeatedly
☒	☐	The statistical test(s) used AND whether they are one- or two-sided<br>*Only common tests should be described solely by name; describe more complex techniques in the Methods section.*
☒	☐	A description of all covariates tested
☒	☐	A description of any assumptions or corrections, such as tests of normality and adjustment for multiple comparisons
☒	☐	A full description of the statistical parameters including central tendency (e.g. means) or other basic estimates (e.g. regression coefficient) AND variation (e.g. standard deviation) or associated estimates of uncertainty (e.g. confidence intervals)
☒	☐	For null hypothesis testing, the test statistic (e.g. *F*, *t*, *r*) with confidence intervals, effect sizes, degrees of freedom and *P* value noted<br>*Give P values as exact values whenever suitable.*
☒	☐	For Bayesian analysis, information on the choice of priors and Markov chain Monte Carlo settings
☒	☐	For hierarchical and complex designs, identification of the appropriate level for tests and full reporting of outcomes
☒	☐	Estimates of effect sizes (e.g. Cohen's *d*, Pearson's *r*), indicating how they were calculated

Our web collection on statistics for biologists contains articles on many of the points above.

Software and code

Policy information about availability of computer code

Data collection	Soilgrids data were downloaded using Google Earth Engine.
Data analysis	Analysis was performed with R (Version 4.1.3) in RStudio (Version 2022.12.0+353). The spatial resolution of the earthworm density layer was altered using Matlab R2020a. Code is available at Zenodo at the following URL/DOI: 10.5281/zenodo.8235224.

For manuscripts utilizing custom algorithms or software that are central to the research but not yet described in published literature, software must be made available to editors and reviewers. We strongly encourage code deposition in a community repository (e.g. GitHub). See the Nature Portfolio guidelines for submitting code & software for further information.

Data

Policy information about availability of data

All manuscripts must include a data availability statement. This statement should provide the following information, where applicable:

- Accession codes, unique identifiers, or web links for publicly available datasets
- A description of any restrictions on data availability
- For clinical datasets or third party data, please ensure that the statement adheres to our policy

All data and code is available at the following URL/DOI: 10.5281/zenodo.8235224, or is already published and publicly available (see references cited in the methods section).

Human research participants

Policy information about studies involving human research participants and Sex and Gender in Research.

Reporting on sex and gender	N/A
Population characteristics	N/A
Recruitment	N/A
Ethics oversight	N/A

Note that full information on the approval of the study protocol must also be provided in the manuscript.

Field-specific reporting

Please select the one below that is the best fit for your research. If you are not sure, read the appropriate sections before making your selection.

☐ Life sciences ☐ Behavioural & social sciences ☒ Ecological, evolutionary & environmental sciences

For a reference copy of the document with all sections, see nature.com/documents/nr-reporting-summary-flat.pdf

Ecological, evolutionary & environmental sciences study design

All studies must disclose on these points even when the disclosure is negative.

Study description	This study combines global data layers on earthworm abundance, soil properties, fertilizer N additions, and crop yields together with earthworm-yield responses from the literature to estimate the impacts of earthworms on global production of key crops.
Research sample	We considered global grid cells from the Monfreda et al. (2000) database that contained key grain and legumes crops considered in our analysis
Sampling strategy	This study is global in nature and sought to include all relevant data that met our criteria (i.e., located in a grid cell with the grain and legumes crops considered in our analysis).
Data collection	We collected global data sets on earthworm abundance, soil properties, fertilizer N additions, and crop yields and upscaled them to 5 arcminute resolution, as needed.
Timing and spatial scale	Data sets included here pull from a variety of sources. We sought to include the most up-to-date datasets that also met the needs of our analysis.
Data exclusions	Grid cells that did not include key grain and legume crops were not included in our analysis
Reproducibility	We provide references to all of the global datasets and can help obtain access to these if needed. Code used for combining geographic data layers is available on Zenodo.
Randomization	This study is global in nature and sought to include all relevant data that met our criteria, thus randomization was not required.
Blinding	We do not believe this to be relevant to our study.

Did the study involve field work? ☐ Yes ☒ No

Reporting for specific materials, systems and methods

We require information from authors about some types of materials, experimental systems and methods used in many studies. Here, indicate whether each material, system or method listed is relevant to your study. If you are not sure if a list item applies to your research, read the appropriate section before selecting a response.

Materials & experimental systems

n/a	Involved in the study
☒	☐ Antibodies
☒	☐ Eukaryotic cell lines
☒	☐ Palaeontology and archaeology
☒	☐ Animals and other organisms
☒	☐ Clinical data
☒	☐ Dual use research of concern

Methods

n/a	Involved in the study
☒	☐ ChIP-seq
☒	☐ Flow cytometry
☒	☐ MRI-based neuroimaging
